# Comparison of secretome from osteoblasts derived from sclerotic versus non-sclerotic subchondral bone in OA: A pilot study

**DOI:** 10.1371/journal.pone.0194591

**Published:** 2018-03-16

**Authors:** Christelle Sanchez, Gabriel Mazzucchelli, Cécile Lambert, Fanny Comblain, Edwin DePauw, Yves Henrotin

**Affiliations:** 1 Bone and Cartilage Research Unit, Arthropôle Liège, University of Liège, Liège, Belgium; 2 Laboratory of Mass Spectrometry—MolSys, Department of Chemistry, University of Liege, Liege, Belgium; Universite de Nantes, FRANCE

## Abstract

**Objective:**

Osteoarthritis (OA) is characterized by cartilage degradation but also by other joint tissues modifications like subchondral bone sclerosis. In this study, we used a proteomic approach to compare secretome of osteoblast isolated from sclerotic (SC) or non sclerotic (NSC) area of OA subchondral bone.

**Design:**

Secretome was analyzed using differential quantitative and relative label free analysis on nanoUPLC G2 HDMS system. mRNA of the more differentially secreted proteins were quantified by RT-PCR in cell culture from 5 other patients. Finally, osteomodulin and fibulin-3 sequences were quantified by western blot and immunoassays in serum and culture supernatants.

**Results:**

175 proteins were identified in NSC osteoblast secretome. Data are available via ProteomeXchange with identifier PXD008494. Compared to NSC osteoblast secretome, 12 proteins were significantly less secreted (Osteomodulin, IGFBP5, VCAM-1, IGF2, 78 kDa glucose-regulated protein, versican, calumenin, IGFBP2, thrombospondin-4, periostin, reticulocalbin 1 and osteonectin), and 13 proteins were significantly more secreted by SC osteoblasts (CHI3L1, fibulin-3, SERPINE2, IGFBP6, SH3BGRL3, SERPINE1, reticulocalbin3, alpha-2-HS-glycoprotein, TIMP-2, IGFBP3, TIMP-1, SERPINF1, CSF-1). Similar changes in osteomodulin, IGF2, SERPINE1, fibulin-3 and CHI3L1 mRNA levels were observed. ELISAs assays confirm the decrease by half of osteomodulin protein in SC osteoblasts supernatant compared to NSC and in OA patients serum compared to healthy subjects. Fibulin-3 epitopes Fib3-1, Fib3-2 and Fib3-3 were also increased in SC osteoblasts supernatant compared to NSC.

**Conclusions:**

We highlighted some proteins differentially secreted by the osteoblasts coming from OA subchondral bone sclerosis. These changes contribute to explain some features observed in OA subchondral bone, like the increase of bone remodeling or abnormalities in bone matrix mineralization. Among identified proteins, osteomodulin was found decreased and fibulin-3 increased in serum of OA patients. These findings suggest that osteomodulin and fibulin-3 fragments could be biomarkers to monitor early changes in subchondral bone metabolism in OA.

## Introduction

Osteoarthritis (OA) is the most common joint disease and this is a major cause of joint pain and disability in the aging population. Its etiology is multifactorial (i.e., age, obesity, joint injury, genetic predisposition), and the pathophysiologic process affects the entirety of the joint [1].

Although it is not yet clear if it precedes or occurs subsequently to cartilage damage, subchondral bone sclerosis is an important feature in OA pathophysiology [2]. It is characterized by local bone resorption and the accumulation of weakly mineralized osteoid substance [3]. Subchondral bone sclerosis is suspected to be linked to cartilage degradation, not only by modifying the mechanical stresses transmitted to the cartilage, but also by releasing biochemical factors with an activity on cartilage metabolism [4–6]. We have previously demonstrated that osteoblasts isolated from subchondral OA bone exhibited an altered phenotype. More precisely, we showed that osteoblasts coming from the thickening (called sclerotic, SC) of subchondral bone located just below a cartilage lesion produced higher levels of alkaline phosphatase, interleukin (IL)-6, IL-8, prostaglandin E_2_, vascular endothelial growth factor (VEGF), matrix metalloproteinase (MMP)-9 and transforming growth factor (TGF)-β1 and type I collagen than osteoblasts coming from the non-thickening neighboring area (called non-sclerotic area, NSC) [7, 8].

To compare secretome of cells living in different *in vivo* conditions is useful, not only to better understand the pathological mechanisms underlying changes in OA subchondral bone, but also to identify soluble biomarkers potentially reflecting these changes.

Using our well-characterized human subchondral osteoblast culture model [7–9], we compared the secretome of osteoblasts coming from sclerotic and non sclerotic OA subchondral bone. This approach allowed to identify changes in secretome that contribute to explain some subchondral bone abnormalities in OA and to propose osteomodulin and fibulin-3 as potential biomarkers of OA subchondral bone remodelling.

## Materials and methods

### Subchondral osteoblasts cell culture

All tissues used in this study were obtained after approval by the faculty of medicine ethics Committee of the University of Liège (number B70720108313, reference 2010/43) and written informed consent was obtained for each subjects. Tibial subchondral bone plates were obtained from 10 OA men undergoing total knee replacement (TKR) surgery. Upon dissection, the femoral, tibial and patellar articular surfaces were evaluated for the pathological cartilage modifications according to the Collins diagram [10]. All cartilage samples showed typical OA lesions, all suffering of grade IV of OA. Patient characteristics are listed in Table 1. Five patients were used for mass spectrometry-based identification and 5 others patients for confirmatory analysis of selected proteins using western blot and immunoassays. The age of the patients ranged from 42 to 83 years (median 70 years). Bone explants were prepared as previously described [7, 8] http://dx.doi.org/10.17504/protocols.io.mkmc4u6. Briefly, after careful elimination of trabecular bone and articular cartilage, OA subchondral bone was dissected to separate NSC from SC zones. We have considered as SC bone only the subchondral bone zones with a thickness greater than 2 mm and either denuded or overlaid by fibrillated cartilage. Also, we have considered as NSC bone only the subchondral bone zones with a maximal thickness of 1 mm [7]. Osteoblasts from SC or NSC subchondral bone were then obtained by outgrowth from explants. At confluence, primary cells were collected by trypsinization, seeded (20,000 cells/cm^2^) in 12-well plates (12-well companion plates, Falcon, BD Biosciences) and grown for 3 days in DMEM containing 10% FBS, 100 U/ml penicillin, 100 μg/ml streptomycin, 10 mM HEPES, 2mM glutamine. The structural difference between SC and NSC bone explant was confirmed histologically as previously reported [7]. Further, before inclusion in the proteomic analysis, phenotype difference between SC and NSC osteoblasts was checked using multiple biomarkers as already published [7, 8] (Table 1).

**Table 1:** Patient characteristics.

N°	Age (Year)	Grade OA	NSC* thickness	SC* thickness	IL-6 SC/NSC	VEGF SC/NSC	Use
SC27	61	IV	0.59–0.65	3.00–4.20	2,5	4	PCR
SC29	68	IV	0.56–0.75	3.48–5.51	5,4	9	Prot/ELISA
SC30	70	IV	0.70–0.87	2.01–2.13	6,3	2,1	Prot/ELISA
SC31	48	IV	0.70–0.81	3.40–6.55	1,8	1,8	Prot/ELISA
SC34	83	IV	0.57–0.72	2.02–2.20	3,8	2,6	Prot/ELISA
SC35	42	IV	0.52–0.89	2.66–3.56	3,3	2,2	Prot/ELISA
SC36	75	IV	0.56–0.65	3.77–5.23	2,4	2	PCR/ELISA/WB
SC38	71	IV	0.51–0.55	2.32–3.70	2,3	1,8	PCR/ELISA/WB
SC39	76	IV	0.78–0.84	2.13–2.15	2,9	2,2	PCR/ELISA
SC40	66	IV	0.42–0.81	2.78–3.10	3,6	3,2	PCR/ELISA/WB

*Thickness range in mm.

Prot = proteomic MS/MS study.

For the experimentations, osteoblasts were rinsed and then cultured in a BSA/FBS free medium for 72 hours. The nutrient media used was DMEM supplemented with 1% ITS (Lonza, Belgium), 10 mM HEPES, 100 U/ml penicillin, 100 μg/ml streptomycin, 2mM glutamine (Lonza, Belgium), 50 μg/ml ascorbic acid (Sigma-Aldrich, Belgium), 20 μg/ml proline (Invitrogen, Belgium). ITS is a premixed cell growth system containing in one ml: 0.625 mg insulin, 0.625 mg transferrin, 0.625 μg selenious acid. These conditioned 72h supernatants were used to perform the secretome analysis. Culture supernatants conditioned during three days by NSC or SC osteoblasts were analyzed by LC-ESI-MS/MS, western blot or immunoassays. Cells were collected and RNA extraction and RT-PCR analysis.

### Proteomic analysis

We performed a proteomic analysis of NSC or SC osteoblasts conditioned culture medium, using differential quantitative and relative label free analysis on nano 2D UPLC- SYNAPT Synapt HDMS G2 HDMS system (Waters, Manchester, UK). Matched NSC and SC culture supernatants of five patients were analyzed.

#### Protein identification by LC-ESI-MS/MS

The samples were reduced, alkylated, concentrated using Amicon (Millipore) with membrane cut-off of 3kDa. The protein content of the samples was then quantified using RCDC kit (Biorad). Aliquotes of 15 μg for each sample were then purified by precipitation using the 2D-Clean up kit (GE) according to manufacturer recommendations, to eliminate impurities not compatible with mass spectrometry analysis. The protein pellets after the washing steps were further resolubilized in bicarbonate ammonium 50mM. The samples were digested in solution with trypsin (16 hours at 37°C ratio tryspin/total proteins (W/W) (1/50), 3h at 37°C with ratio 1/100 in 80% ACN). The reaction was stopped by addition of formic acid. The samples were evaporated to dryness in a speed vacuum.

The samples were dissolved in water 0.1% formic acid then an aliquote corresponding to 3.5 μg of protein digest for each sample was purified using a Zip-Tip C18 High Capacity according to manufacturer recommendations. The samples were evaporated to dryness in a speed vacuum.

Digested samples were analyzed (separately) using the 2D-nano-Acquity® UPLC system (Waters, Manchester, UK) coupled with the SYNAPT G2 HDMS Mass Spectrometer (Waters, Manchester, UK). 3.5 μg of lyophilized peptides were dissolved in 12.5 μL of 100mM ammonium formiate buffer (pH10). Samples were spiked with MassPREP™ Digestion Standard Mixtures 1 or 2 (IS1 and IS2) containing an equimolar or different amounts of yeastalcohol dehydrogenase, rabbit glycogen phosphorylase beta, bovine serum albumin and yeast enolase (Waters, Manchester). Finlay, 9 μL of the sample containing 150 fmoles of yeast alcohol dehydrogenase was injected, corresponding to an estimated sample load of 2.5μg.

#### PLGS analysis

The LC-MSMS analyses and the bioinformatic processing were performed as previously described by Baiwir et al. [11]. Protein identification was performed using the data base extracted from UNIPROT Sus Crofa with manual addition of the protein sequences used as spikes and internal standards: MPDS mix.

The PLGS score is a probabilistic score correlated to the degree of confidence regarding the identification of a protein. Therefore a high value of PLGS score correlates with the fact that a high quality and quantity of information was observed by MS and MSE. The score is attributed to the protein after database search. This database search involves also the search on a randomized database recomputed from the original data base to evaluate the risk of false positive protein identification.

For identification, the minimum to consider is at least two different peptides per protein identified and to check the false positive rate, this should be as low as possible (the false positive rate will be of maximum 4% because of the settings used in PLGS database search).

The mass spectrometry proteomics data have been deposited to the ProteomeXchange Consortium via the PRIDE partner repository with the dataset identifier PXD008494 [12].

### Quantitative Real-time RT PCR

RNA from 1.10^6^ cells was isolated by RNeasy mini kit total RNA isolation system (Qiagen, Belgium) and polymerase chain reaction (PCR) was performed by using the Rotor Gene (Qiagen, Belgium)- SYBR premix Ex Taq (Takara, Belgium). The PCR template source was either 3 ng first-strand cDNA or purified DNA standard. PCR program comprised an initial denaturation step at 95°C for 10 s followed by 40 cycles of denaturation at 95xC for 5 s and annealing/extension at 60°C for 25 s and an ending melting step from 65°C to 96°C with a 1°C increase each s. The following primer sequences were used to amplify the desired cDNA: HPRT forward 5’-TGTAATGACCAGTCAACAGGG-3' and reverse 5’-TGCCTGACCAAGGAAAGC-3’, CHI3L1 forward 5’- CTACCCTGGACGGAGAGACA-3’ and reverse 5’- GGACTTGCATCCTCCTGACC-3’, SH3 domain binding glutamic acid rich-like protein 3 forward 5’- CTGAGGGATGAGATGCGAG-3’ and reverse 5’- CTCTGGACAGGCTTGACT-3’, SERPTIN E1 forward 5’- ATGCCCTCTACTTCAACGGC-3’ and reverse 5’- TTCCAGTGGCTGATGAGCTG-3’, SERPIN E2 forward 5’- TCCAGCCTCTGCCTGTGATT-3’ and reverse 5’- AATACACTGCGTTGACGAGGA-3’, periostin forward 5’- CTGGCTGGAAAACAGCAAACC-3’ and reverse 5’- CGGAATATGTGAATCGCACCG-3’, IGFBP5 forward 5’- GTCCAAGTTTGTCGGGGGAG-3’ and reverse 5’- AAGTCCCCGTCAACGTACTC-3’, IGFBP6 forward 5’- CATCCGCCCAAGGACGAC-3’ and reverse 5’- GCCTGCTTGGGGTTTACTCTC-3’, calumenin forward 5’- GCGACAGTTTCTTATGTGCCT-3’ and reverse 5’- AAAGGTCTTTGCTTCTTCAGCAC-3’, fibulin-3 forward 5’-CCTCAAGCTACCTGTGTCAATA-3’ and reverse 5’-GGATTTCGTGGATAACAACGG-3’, OMD forward 5’- TCCTGGTTTGCCTTCTTCACTT-3’ and reverse 5’- GGGTCAATAGAAGGACACATCAC-3’, alpha-2-HS-glycoprotein forward 5’-GCTCAGAACAACGGCTCCAA-3’ and reverse 5’-GGTTACACTTGGCTGCCTCT-3’, VCAM1 forward 3’-AATGGAAGATTCTGGGGT-5’ and reverse 3’-TGTGTCTCCTGTCTCCG-5’, CSF1 forward 3’-TGGCGAGCAGGAGTATC-5’ and reverse 3’-ACCAGGAGAAATGCCTT-5’, IGF2 forward 3’-TGCTTCTCACCTTCTTGG-5’ and reverse 3’-CACTCCTCAACGATGCC-5’. Hypoxanthine-guanine phosphoribosyltransferase (HPRT) was used as an internal standard and the ratio of genes to HPRT were calculated. After HPRT normalization for each gene, relative expression of SC to NSC was calculated. Five patients were analyzed

### Western blotting

Serum of 12 men were used in western-blotting experiments, 6 healthy (mean age 43, range 22–61) and 6 suffering of severe OA just before TKR (mean age 67, range 44–82). Before experiment serum were depleted in IgG and albumin using ProteoPrep kits (Sigma). During this process serum is diluted by approximately 3.3-fold. Supernatants of three independent Subchondral osteoblast culture were 20-fold concentrated using Amicon Ultra 3kDa 2 ml column (Millipore). Recombinant human OMD (R&D systems, 2884-AD) was used as positive control. Depleted serum (15μl) or osteoblasts concentrated supernatant (6 μl = 6 μg total proteins) were fractioned by electrophoresis on a polyacrylamide gel (9%) and transferred onto a polyvinylidene difluoride membrane. Membranes were blocked overnight 4°C with Roche Blocking Reagent (Villevorde, Belgium). Membranes were then incubated overnight at 4°C with a biotinylated polyclonal goat antiserum, affinity purified, raised against the whole osteomodulin protein (BAF2884, R&D systems, Minneapolis, USA) 1:200 dilution in 0.5% Roche blocking reagent. Streptavidin-Horse-radish peroxidase (HRP) (1:2500 dilution) was used as detection (Roche). The reaction was revealed with Luminata classico Western blotting substrate (Millipore) and capture with an ImageQuantLAS4000 (Amersham).

### OMD1 and OMD2 ELISA

BalbC mice were immunized against 2 peptides of osteomodulin, OMD1 ^148^LEHNNLEEFPFPLPK^162^ or OMD2 ^261^LRMSHNKLQDIPYNI^276^ -KLH coupled peptides. Antisera were specific to these sequences and do not cross with 60% homolog peptides contained in the Lumican protein. Assays were made using biotinylated peptides, without the cysteine added for the KLH coupling, fixed on a streptavidin-coated 96-wells-plate. The competitive ELISA was carried using 50 μl of unbiotinylated and uncoupled peptide as competitor, with a standard curve concentrations comprise between 520 nM and 8 nM, and 50 μl of OMD antisera, 1h at room temperature under 500 rpm agitation. OMD1 antiserum was used at 1:7500 and OMD2 antiserum at 1:1000 dilution. Peroxidase AffiniPure Goat Anti-Mouse IgG, Fcγ Fragment Specific (Jackson ImmunoResearch) was used as secondary antibody (1:10000 dilution). Human sera dilution was shown to be parallel to the standard curve from 1:2 to 1:8 dilution. All sera and reagents were prepared in Diluent Reagent Buffer (D-Tek, Belgium). Assays were performed at 1:3 dilution of the sera for human. Culture supernatants were assayed undiluted.

### Fib3-1, Fib3-2 and Fib3-3 ELISA

Three fibulin-3 peptides were determined. The sequence of Fib3-1, Fib3-2 and Fib3-3 were ^331^TCQDINECETTNECR^345^, ^377^CVCPVSNAMCR^387^ and ^430^SGNENGEFYLR^440^, respectively. They were quantified in triplicate by specific competitive Enzyme-Linked Immunosorbent Assays (ELISAs) (Artialis SA, Liège, Belgium), as previously described [13].

### Statistical analysis

For proteomic, statistical analysis was made with ProteinLynx Global SERVER vs2.5 includes identification of the peptides and proteins and their relative quantification on the base of co-analysis of a second internal standard composed of several proteins digests, present in both samples to be compared at different abundance ratio. The linear dynamic range of this label free quantitative technique is around 3 to 4 order of magnitude.

For gene expression and ELISA, the results (mean ± SD) were expressed as HPRT-normalized gene expression or as concentration of protein per μg of DNA. A unpaired t-test was performed for each experiment. For western-blot and ELISA quantification in sera, following a D'Agostino & Pearson omnibus normality test, a Mann-Whitney test or Spearman correlation was performed (GraphPad Prism 6.0).

## Results

### OA osteoblasts secretome

We performed a proteomic analysis of OA osteoblasts conditioned culture medium, using differential quantitative and relative label free analysis on nano2D-UPLC- SYNAPT G2 HDMS system (Waters, Manchester, UK) to identify the differential protein production between NSC and SC osteoblasts. Looking at the global NSC secretome, we used high top 3 analysis to estimate the protein abundance [14]. Without the internal standard and the proteins added in the supernatant during the cell culture (IGF-1 and transferrin), 175 proteins were found in the osteoblast secretome (Table 2). Taking in account the different isoforms of proteins, 318 proteins were found in osteoblasts secretome.

**Table 2:** Estimation of abundance of NSC osteoblasts secreted proteins using high top 3.

Accession	F	Entry	Description	fmol (mean)	fmol (CV)
P02452	5	CO1A1_HUMAN	Collagen alpha-1(I) chain	353,23	0,04
P08123	5	CO1A2_HUMAN	Collagen alpha-2(I) chain	242,46	0,12
P09486	5	SPRC_HUMAN	SPARC	168,46	0,13
Q8NBF2	4	NHLC2_HUMAN	NHL repeat-containing protein 2	166,75	0,08
P02751-5	5	FINC_HUMAN	Isoform Fibronectin (V+I-10)- of Fibronectin	161,06	0,26
P51884	5	LUM_HUMAN	Lumican	142,97	0,21
Q99715-2	5	COCA1_HUMAN	Isoform Short of Collagen alpha-1(XII) chain	124,50	0,51
P04264	5	K2C1_HUMAN	Keratin, type II cytoskeletal 1	113,84	0,71
Q8IXQ4	3	K1704_HUMAN	Uncharacterized protein KIAA1704	102,81	1,41
P35900	4	K1C20_HUMAN	Keratin, type I cytoskeletal 20	83,26	0,21
Q16270	5	IBP7_HUMAN	Insulin-like growth factor-binding protein 7	81,94	0,69
P12109	5	CO6A1_HUMAN	Collagen alpha-1(VI) chain	77,86	0,38
P35527	5	K1C9_HUMAN	Keratin, type I cytoskeletal 9	74,59	1,02
P07585	5	PGS2_HUMAN	Decorin	73,13	0,47
P22692	5	IBP4_HUMAN	Insulin-like growth factor-binding protein 4	72,57	0,22
P21810	5	PGS1_HUMAN	Biglycan	68,45	0,38
P08727	4	K1C19_HUMAN	Keratin, type I cytoskeletal 19	64,75	0,11
Q12841	5	FSTL1_HUMAN	Follistatin-related protein 1	64,25	0,20
P09382	5	LEG1_HUMAN	Galectin-1	63,39	0,11
P02461	5	CO3A1_HUMAN	Collagen alpha-1(III) chain	63,25	0,30
P08670	5	VIME_HUMAN	Vimentin	48,40	0,21
P08253	5	MMP2_HUMAN	72 kDa type IV collagenase	44,77	0,45
P13611-3	5	CSPG2_HUMAN	Isoform V2 of Versican core protein	39,03	0,58
P01033	5	TIMP1_HUMAN	Metalloproteinase inhibitor 1	38,95	0,50
O43852	5	CALU_HUMAN	Calumenin	38,91	0,32
P36222	4	CH3L1_HUMAN	Chitinase-3-like protein 1	35,83	1,06
P12110	5	CO6A2_HUMAN	Collagen alpha-2(VI) chain	35,10	0,74
P13645	5	K1C10_HUMAN	Keratin, type I cytoskeletal 10	34,72	0,35
Q15113	5	PCOC1_HUMAN	Procollagen C-endopeptidase enhancer 1	33,42	0,39
P24593	5	IBP5_HUMAN	Insulin-like growth factor-binding protein 5	31,44	0,55
P26022	5	PTX3_HUMAN	Pentraxin-related protein PTX3	29,86	0,52
P07951-2	4	TPM2_HUMAN	Isoform Epithelial TMe1 of Tropomyosin beta chain	28,97	0,48
P35908	5	K22E_HUMAN	Keratin, type II cytoskeletal 2 epidermal	27,65	0,62
P07996	5	TSP1_HUMAN	Thrombospondin-1	27,64	0,39
P07602	5	SAP_HUMAN	Proactivator polypeptide	26,09	1,13
P24821	3	TENA_HUMAN	Tenascin	25,71	1,27
P62988	5	UBIQ_HUMAN	Ubiquitin	25,52	0,12
P60709	5	ACTB_HUMAN	Actin, cytoplasmic 1	25,30	0,22
P01023	5	A2MG_HUMAN	Alpha-2-macroglobulin	25,10	0,75
P01344	5	IGF2_HUMAN	Insulin-like growth factor II	24,68	0,53
P24821-4	3	TENA_HUMAN	Isoform HT-33 of Tenascin	23,62	0,22
P27482	4	CALL3_HUMAN	Calmodulin-like protein 3	23,23	0,64
P17661	4	DESM_HUMAN	Desmin	22,90	1,01
P02751-6	3	FINC_HUMAN	Isoform Fibronectin (V+III-15)- of Fibronectin	21,26	0,06
Q15063-3	5	POSTN_HUMAN	Isoform 3 of Periostin	20,79	0,30
P26038	5	MOES_HUMAN	Moesin	19,14	1,05
P10124	5	SRGN_HUMAN	Serglycin	18,56	0,41
Q02818	5	NUCB1_HUMAN	Nucleobindin-1	18,20	0,28
P02533	5	K1C14_HUMAN	Keratin, type I cytoskeletal 14	18,08	1,18
P13647	5	K2C5_HUMAN	Keratin, type II cytoskeletal 5	17,90	1,13
P69905	5	HBA_HUMAN	Hemoglobin subunit alpha	17,26	0,16
P02768	4	ALBU_HUMAN	Serum albumin	17,23	1,34
P67936	5	TPM4_HUMAN	Tropomyosin alpha-4 chain	17,06	0,43
P49747	5	COMP_HUMAN	Cartilage oligomeric matrix protein	16,29	1,08
P61769	5	B2MG_HUMAN	Beta-2-microglobulin	15,76	0,27
Q15293	5	RCN1_HUMAN	Reticulocalbin-1	15,47	0,20
O00622	3	CYR61_HUMAN	Protein CYR61	15,41	1,10
P05997	5	CO5A2_HUMAN	Collagen alpha-2(V) chain	14,56	0,27
Q9UBP4	5	DKK3_HUMAN	Dickkopf-related protein 3	14,08	0,40
P05121	5	PAI1_HUMAN	Plasminogen activator inhibitor 1	13,98	0,54
P02545	5	LMNA_HUMAN	Lamin-A/C	13,45	1,68
O95965	5	ITGBL_HUMAN	Integrin beta-like protein 1	12,96	0,31
P62736	3	ACTA_HUMAN	Actin, aortic smooth muscle	12,40	0,64
P07355	5	ANXA2_HUMAN	Annexin A2	12,36	1,49
Q15582	5	BGH3_HUMAN	Transforming growth factor-beta-induced protein ig-h3	12,25	0,66
P09871	5	C1S_HUMAN	Complement C1s subcomponent	12,18	0,46
P36955	5	PEDF_HUMAN	Pigment epithelium-derived factor	12,16	0,82
P20908	5	CO5A1_HUMAN	Collagen alpha-1(V) chain	11,46	0,65
Q08380	5	LG3BP_HUMAN	Galectin-3-binding protein	11,44	0,44
P63104	3	1433Z_HUMAN	14-3-3 protein zeta/delta	10,78	0,80
P15531	5	NDKA_HUMAN	Nucleoside diphosphate kinase A	10,16	1,30
P35442	5	TSP2_HUMAN	Thrombospondin-2	10,13	0,50
P55287	5	CAD11_HUMAN	Cadherin-11	9,81	1,25
P06703	5	S10A6_HUMAN	Protein S100-A6	9,61	0,16
P17936	4	IBP3_HUMAN	Insulin-like growth factor-binding protein 3	9,59	0,64
O43707	5	ACTN4_HUMAN	Alpha-actinin-4	9,34	1,47
P00736	5	C1R_HUMAN	Complement C1r subcomponent	9,01	0,59
Q76M96	5	CCD80_HUMAN	Coiled-coil domain-containing protein 80	8,81	1,62
O00391	5	QSOX1_HUMAN	Sulfhydryl oxidase 1	8,72	0,64
P11216	3	PYGB_HUMAN	Glycogen phosphorylase, brain form	8,27	0,38
Q05707	3	COEA1_HUMAN	Collagen alpha-1(XIV) chain	7,95	0,45
Q9NRN5	5	OLFL3_HUMAN	Olfactomedin-like protein 3	6,85	0,85
P23142	5	FBLN1_HUMAN	Fibulin-1	6,71	0,49
P08572	5	CO4A2_HUMAN	Collagen alpha-2(IV) chain	6,54	0,72
P00338	5	LDHA_HUMAN	L-lactate dehydrogenase A chain	6,50	0,76
P11021	4	GRP78_HUMAN	78 kDa glucose-regulated protein	6,45	1,14
P19320	5	VCAM1_HUMAN	Vascular cell adhesion protein 1	6,23	0,62
Q9Y287	5	ITM2B_HUMAN	Integral membrane protein 2B	6,22	0,24
P41222	3	PTGDS_HUMAN	Prostaglandin-H2 D-isomerase	6,18	0,40
P06753	3	TPM3_HUMAN	Tropomyosin alpha-3 chain	6,03	0,13
Q13822	4	ENPP2_HUMAN	Ectonucleotide pyrophosphatase/phosphodiesterase family member 2	5,78	0,57
Q96D15	5	RCN3_HUMAN	Reticulocalbin-3	5,75	1,32
P62158	5	CALM_HUMAN	Calmodulin	5,66	0,28
P04075	3	ALDOA_HUMAN	Fructose-bisphosphate aldolase A	5,56	0,77
P10599	5	THIO_HUMAN	Thioredoxin	5,39	0,28
P00441	4	SODC_HUMAN	Superoxide dismutase [Cu-Zn]	5,22	0,41
P31431	3	SDC4_HUMAN	Syndecan-4	5,17	0,11
P05155	5	IC1_HUMAN	Plasma protease C1 inhibitor	5,13	0,49
P14618	5	KPYM_HUMAN	Pyruvate kinase isozymes M1/M2	4,98	0,37
Q9H299	5	SH3L3_HUMAN	SH3 domain-binding glutamic acid-rich-like protein 3	4,89	0,16
Q99983	3	OMD_HUMAN	Osteomodulin	4,76	0,22
P18065	5	IBP2_HUMAN	Insulin-like growth factor-binding protein 2	4,64	0,30
Q92743	5	HTRA1_HUMAN	Serine protease HTRA1	4,55	0,34
P35579	3	MYH9_HUMAN	Myosin-9	4,52	0,49
P07942	5	LAMB1_HUMAN	Laminin subunit beta-1	4,45	0,37
Q16363	5	LAMA4_HUMAN	Laminin subunit alpha-4	4,37	0,58
P12814	5	ACTN1_HUMAN	Alpha-actinin-1	4,36	0,42
P80303	5	NUCB2_HUMAN	Nucleobindin-2	4,29	0,29
P62258	5	1433E_HUMAN	14-3-3 protein epsilon	4,17	0,45
P16035	5	TIMP2_HUMAN	Metalloproteinase inhibitor 2	4,10	0,49
O14498	5	ISLR_HUMAN	Immunoglobulin superfamily containing leucine-rich repeat protein	4,04	0,65
P13497	5	BMP1_HUMAN	Bone morphogenetic protein 1	4,00	0,53
P07195	5	LDHB_HUMAN	L-lactate dehydrogenase B chain	3,93	0,82
P11047	5	LAMC1_HUMAN	Laminin subunit gamma-1	3,89	0,33
P23528	3	COF1_HUMAN	Cofilin-1	3,72	0,30
P27797	3	CALR_HUMAN	Calreticulin	3,60	0,22
Q01995	5	TAGL_HUMAN	Transgelin	3,57	0,48
Q9Y6C2	5	EMIL1_HUMAN	EMILIN-1	3,51	0,46
O94985	5	CSTN1_HUMAN	Calsyntenin-1	3,46	0,34
P05452	3	TETN_HUMAN	Tetranectin	3,45	0,79
Q08431	5	MFGM_HUMAN	Lactadherin	3,45	0,69
Q14766	5	LTBP1_HUMAN	Latent-transforming growth factor beta-binding protein 1	3,43	0,26
P02538	4	K2C6A_HUMAN	Keratin, type II cytoskeletal 6A	3,41	1,01
Q6FHJ7	4	SFRP4_HUMAN	Secreted frizzled-related protein 4	3,36	0,53
Q68BL8	5	OLM2B_HUMAN	Olfactomedin-like protein 2B	3,23	0,21
O76076	3	WISP2_HUMAN	WNT1-inducible-signaling pathway protein 2	3,23	0,25
Q9BRK5	5	CAB45_HUMAN	45 kDa calcium-binding protein	3,22	0,15
P07737	3	PROF1_HUMAN	Profilin-1	3,17	0,52
P24592	5	IBP6_HUMAN	Insulin-like growth factor-binding protein 6	3,16	0,34
Q14112	4	NID2_HUMAN	Nidogen-2	3,12	0,21
P07237	5	PDIA1_HUMAN	Protein disulfide-isomerase	3,10	0,15
P14543	5	NID1_HUMAN	Nidogen-1	3,04	0,24
P60174	4	TPIS_HUMAN	Triosephosphate isomerase	2,95	0,51
P62805	4	H4_HUMAN	Histone H4	2,94	0,54
P29279	3	CTGF_HUMAN	Connective tissue growth factor	2,81	0,91
P06396	4	GELS_HUMAN	Gelsolin	2,81	0,71
O95967	5	FBLN4_HUMAN	EGF-containing fibulin-like extracellular matrix protein 2	2,79	0,39
P23284	3	PPIB_HUMAN	Peptidyl-prolyl cis-trans isomerase B	2,78	0,37
Q08629	3	TICN1_HUMAN	Testican-1	2,73	0,41
P50454	5	SERPH_HUMAN	Serpin H1	2,72	0,62
P55290	4	CAD13_HUMAN	Cadherin-13	2,58	0,44
O95084	4	PRS23_HUMAN	Serine protease 23	2,56	0,38
P07858	4	CATB_HUMAN	Cathepsin B	2,51	0,35
P60660	4	MYL6_HUMAN	Myosin light polypeptide 6	2,50	0,26
P81605	3	DCD_HUMAN	Dermcidin	2,44	0,45
Q8NBJ4	5	GOLM1_HUMAN	Golgi membrane protein 1	2,42	0,83
Q05682	5	CALD1_HUMAN	Caldesmon	2,42	0,99
P01210	4	PENK_HUMAN	Proenkephalin-A	2,40	0,38
P09493	4	TPM1_HUMAN	Tropomyosin alpha-1 chain	2,40	0,44
P04406	4	G3P_HUMAN	Glyceraldehyde-3-phosphate dehydrogenase	2,37	0,29
Q14767	3	LTBP2_HUMAN	Latent-transforming growth factor beta-binding protein 2	2,37	0,39
Q8IUX7	5	AEBP1_HUMAN	Adipocyte enhancer-binding protein 1	2,35	0,52
P07339	4	CATD_HUMAN	Cathepsin D	2,33	0,45
Q06828	3	FMOD_HUMAN	Fibromodulin	2,32	0,26
P35052	3	GPC1_HUMAN	Glypican-1	2,26	0,38
P04259	5	K2C6B_HUMAN	Keratin, type II cytoskeletal 6B	2,19	0,33
Q06830	3	PRDX1_HUMAN	Peroxiredoxin-1	2,18	0,21
Q6EMK4	3	VASN_HUMAN	Vasorin	2,17	0,26
Q15063	5	POSTN_HUMAN	Periostin	2,10	0,54
Q16610	5	ECM1_HUMAN	Extracellular matrix protein 1	2,08	0,36
P29401	4	TKT_HUMAN	Transketolase	1,92	0,47
P61981	3	1433G_HUMAN	14-3-3 protein gamma	1,86	0,44
P49746	5	TSP3_HUMAN	Thrombospondin-3	1,66	0,35
Q02809	4	PLOD1_HUMAN	Procollagen-lysine,2-oxoglutarate 5-dioxygenase 1	1,64	0,50
P68104	5	EF1A1_HUMAN	Elongation factor 1-alpha 1	1,64	0,55
P07093	4	GDN_HUMAN	Glia-derived nexin	1,50	1,20
P08238	3	HS90B_HUMAN	Heat shock protein HSP 90-beta	1,36	0,25
P11142	3	HSP7C_HUMAN	Heat shock cognate 71 kDa protein	1,34	0,52
Q9Y240	3	CLC11_HUMAN	C-type lectin domain family 11 member A	1,30	0,12
P13646	3	K1C13_HUMAN	Keratin, type I cytoskeletal 13	1,22	0,61
P17931	3	LEG3_HUMAN	Galectin-3	1,21	0,30
Q12805	5	FBLN3_HUMAN	EGF-containing fibulin-like extracellular matrix protein 1	1,06	1,45
P18206	4	VINC_HUMAN	Vinculin	1,04	0,62
P06737	3	PYGL_HUMAN	Glycogen phosphorylase, liver form	0,79	0,27

F = frequency, occurrence in the 5 patients.

The more abundant secreted proteins were type I collagen, SPARC (osteonectin), fibronectin and lumican. Other collagens were present (type XII, VI, II, V and XIV follow their abundance), and insulin growth factor binding proteins (IGFBPs) were particularly abundant (IGFBP7, 4, 5, 3, 2 and 6 according to their abundance).

### Differentially secreted proteins between NSC and SC osteoblasts

Twelve proteins were found less secreted by SC than NSC osteoblasts (Osteomodulin (OMD), IGFB5, vascular cell adhesion molecule (VCAM)1, IGF2, 78 kDa glucose-regulated protein, versican, calumenin, IGFBP2, thrombospondin-4, periostin, reticulocalbin 1 and SPARC, Table 3). OMD level was particularly lowered in SC osteoblasts supernatant compared to NSC. More precisely, OMD was found to be reduced by 54% in two patients (p = 0.0212) and undetectable in the three others SC osteoblasts culture.

**Table 3:** MS/MS proteomic analysis of osteoblasts secretome.

Decreased in SC (ratio SC/NSC)	Increased in SC (ratio SC/NSC)	NSC only	SC only
SPARC (0.8, p<0.001, n = 5)	SERPIN F1 (1.3, p = 0.01, n = 5)		CSF1 (n = 3)
Reticulocalbin 1 (0.7, p = 0.01, n = 4)	TIMP-1 (1.4, p<0.001, n = 5)		
Periostin (0.7, p<0.001, n = 5)	IGFBP3 (1.5, p<0.001, n = 4)		
TSP4 (0.6, p = 0.04, n = 3)	TIMP-2 (1.7, p = 0.01, n = 4)		
IGFBP2 (0.6, p = 0.01, n = 4)	Alpha-2-HS-glycoprotein (1.9, p<0.001, n = 3)		
Calumenin (0.6, p<0.001, n = 5)	Reticulocalbin3 (1.8, p = 0.01, n = 5)		
Versican (0.6, p<0.001, n = 5)	SERPIN E1 (1.9, p<0.001, n = 5)		
78 kDa glucose-regulated protein (0.5, p<0.001, n = 4)	SH3BGRL3 (1.9, p<0.001, n = 5)		
IGF2 (0.5, p<0.001, n = 4)	IGFBP6 (2, p<0.001, n = 5)		
VCAM1 (0.4, p<0.001, n = 5)	SERPIN E2 (2.4, p<0.001, n = 3)		
Osteomodulin (0.4, p = 0.021, n = 5)	Fibulin-3 (2.5, p<0.001, n = 5)		
IGFBP5 (0.4, p<0.001, n = 5)	CHI3L1 (7.1, p<0.001, n = 4)		

CHI3L1: chitinase-3-like protein 1; CSF1: macrophage colony-stimulating factor; IGF: insulin growth factor; IGFBP: IGF binding protein; SERPIN: serine peptidase inhibitor; SH3BGRL3: SH3 domain-binding glutamic acid-rich-like protein 3; SPARC: secreted protein, acidic, cysteine-rich (osteonectin); TIMP: tissue inhibitor of metalloproteinase; TSP: thrombospondin, VCAM: vascular cell adhesion molecule. Cut off: ratio SC/NSC <0.8 or >1.2. NSC only or SC only in some patients are considered as a decrease or an increase.

Conversely, 12 proteins are found to be in higher concentration in SC than NSC osteoblasts secretome (chitinase 3 like protein 1 (CHI3L1), fibulin-3, SERPINE2, IGFBP6, SH3 domain-binding glutamic acid-rich-like protein 3 (SH3BGRL3), SERPINE1, reticulocalbin3, alpha-2-HS-glycoprotein, tissue inhibitor of metalloproteases (TIMP)-2, IGFBP3, TIMP-1, SERPINF1). Macrophage colony-stimulating factor (CSF1) was only found in SC osteoblasts secretome in three patients (Table 3).

### Gene expression of differentially secreted proteins in NSC and SC osteoblasts

We analyzed the mRNA levels of fourteen significantly differentially secreted proteins: OMD, periostin, IGF2, IGFBP5, calumenin and VCAM1 for the lowered and fibulin-3, SERPIN E1 and E2, IGFBP6, SH3BGRL3, alpha-2-HS-glycoprotein, CHI3L1 and CSF1, for the increased. Alpha-2-HS-glycoprotein mRNA levels was undetectable in our experimental conditions.

**Fig 1:**
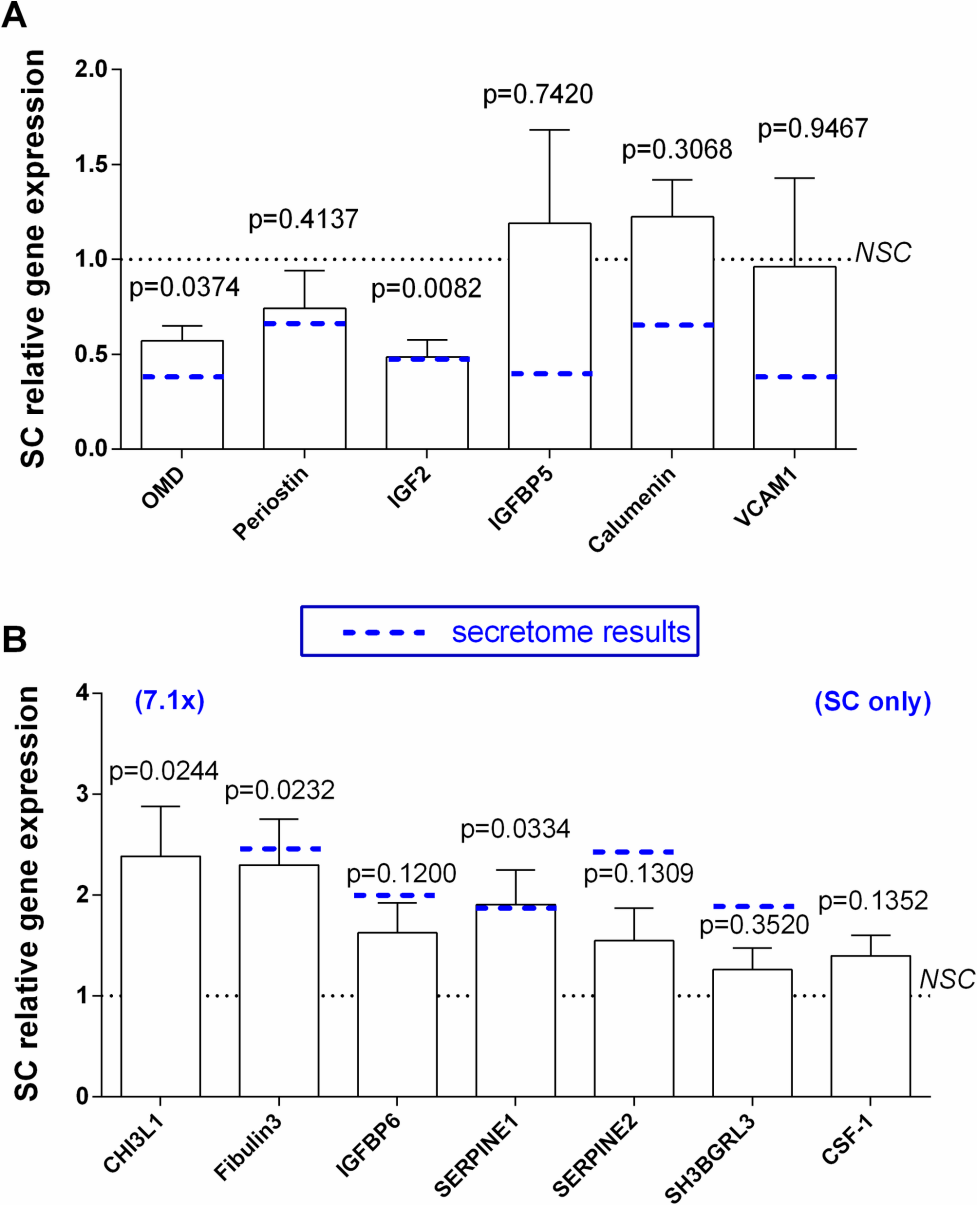
Gene expression by NSC or SC osteoblasts. (A) less secreted proteins (B) more secreted proteins. The mRNA copy numbers were normalized against the corresponding copy number of HPRT mRNA and results are showed as the SC relative to NSC gene expression (mean ± SEM of five independent experiments performed with bone biopsy coming from different donors). In each experiment, each experimental condition was performed in duplicate. P: comparisons of mean values between NSC and SC were performed by unpaired t-test. Dotted blue line in each gene represents the result obtained with MS/MS in secretome with the corresponding protein.

Out of the six decreased proteins, only OMD (0.57 +/- 0.19 fold, p = 0.0374) and IGF2 (0.49 +/- 0.15 fold, p = 0.0082) mRNA levels were also lower in SC than in NSC osteoblasts in the 5 independent cultures (Fig 1A). Contrariwise, mRNA levels of periostin, calumenin, VCAM1 and IGFBP5 didn’t significantly vary between NSC and SC osteoblasts (Fig 1).

Out of the eight more increased proteins, CHI3L1 (2.42 +/- 1.13 fold, p = 0.0244), fibulin-3 (2.22 +/- 0.50, p = 0.0232), and SERPIN E1 (1.90+/-0.76 fold, p = 0.0334) mRNA levels were higher in SC than in NSC osteoblasts (Fig 1). SERPIN E2, IGFBP6, CSF-1 and SH3BGRL3 gene expression didn’t significantly vary between NSC and SC cells (Fig 1).

### Quantification of osteomodulin in osteoblasts supernatant and human serum

**Fig 2:**
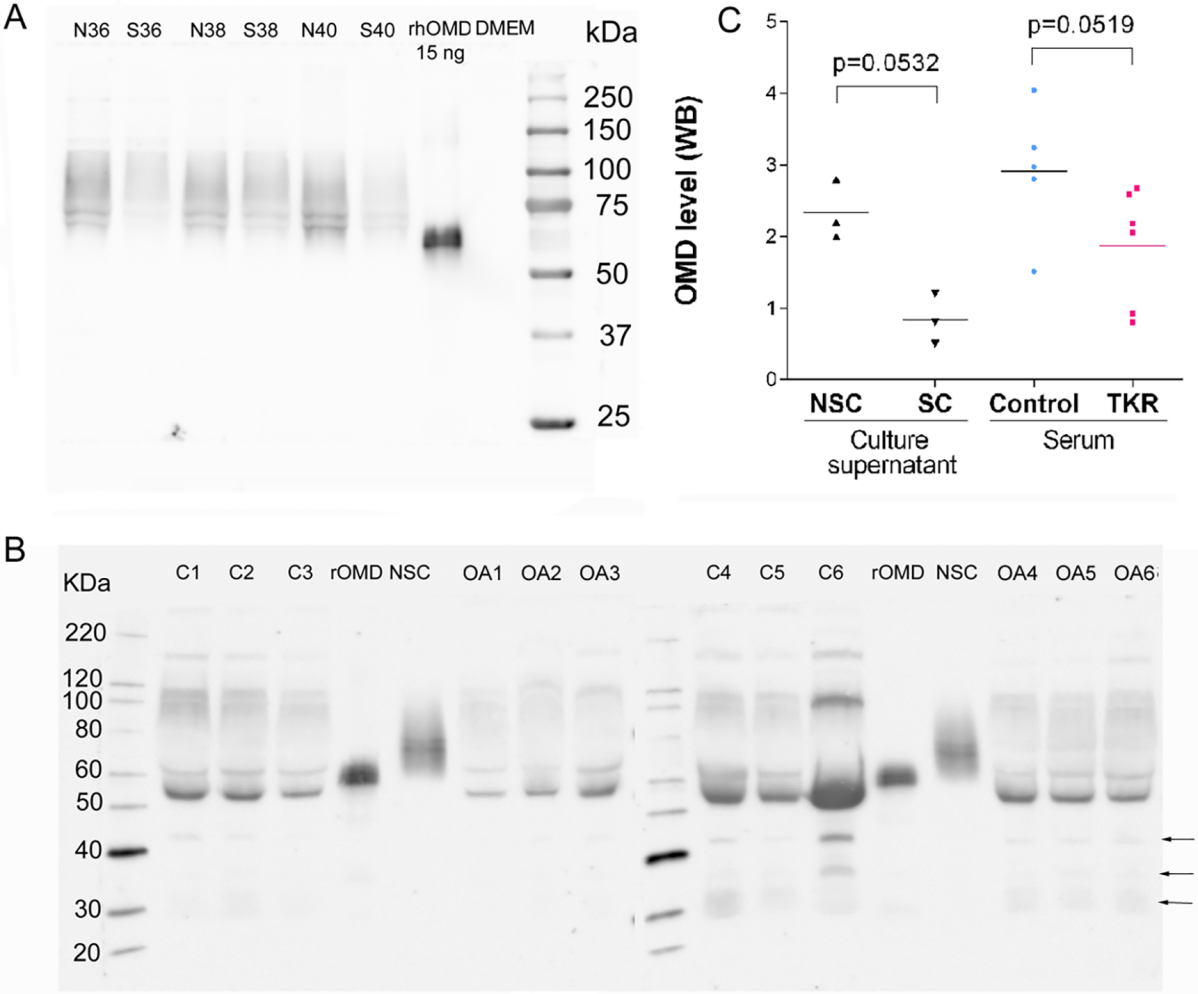
Western-blotting detection of osteomodulin using goat polyclonal antisera raised against entire osteomodulin. (A)(B) rhOMD: human recombinant osteomodulin (positive control), DMEM: concentration unconditioned culture supernatant (negative control), NSC: non sclerotic osteoblasts supernatant (N36, N38, N40), SC: sclerotic osteoblasts supernatant (S36, S38, S40), C1-6: six healthy patients albumin/IgG depleted serum, OA1-6: six OA patients before total knee replacement surgery albumin/IgG depleted serum. (C) Quantification of osteomodulin western blot performed on osteoblasts culture supernatants and on albumin/IgG depleted serum of 6 healthy patients (controls) and 6 patients before total knee replacement (OA). Quantification was made using ImageJ on total band and normalized with total protein charged visualized with reverse total protein stain (Pierce).

Because OMD was the most diminished in SC osteoblasts secretome, we also investigated this protein by western blot using a goat polyclonal antiserum raised against the entire OMD protein (R&D systems), both in osteoblasts culture supernatants and human serum (Fig 2). The recombinant OMD (positive control) was made in a mouse myeloma cell line and runs between 60-66kDa on reducing gels.

In osteoblasts culture supernatants, OMD appeared in two major bands of approximately 70 and 75 kDa. OMD was less abundant in SC than in NSC conditioned culture medium (Fig 2A).

In human serum, antibodies bound mainly a 54 kDa OMD form. Minor 42, 35 and 30 kDa forms were also observed (Fig 2B).

We have quantified OMD bands in serum of 6 healthy patients and 6 severe OA patients just before total knee replacement (TKR). Normalized signal was reduced by half in TKR compared to healthy patients (p = 0.0519, Fig 2C).

**Fig 3:**
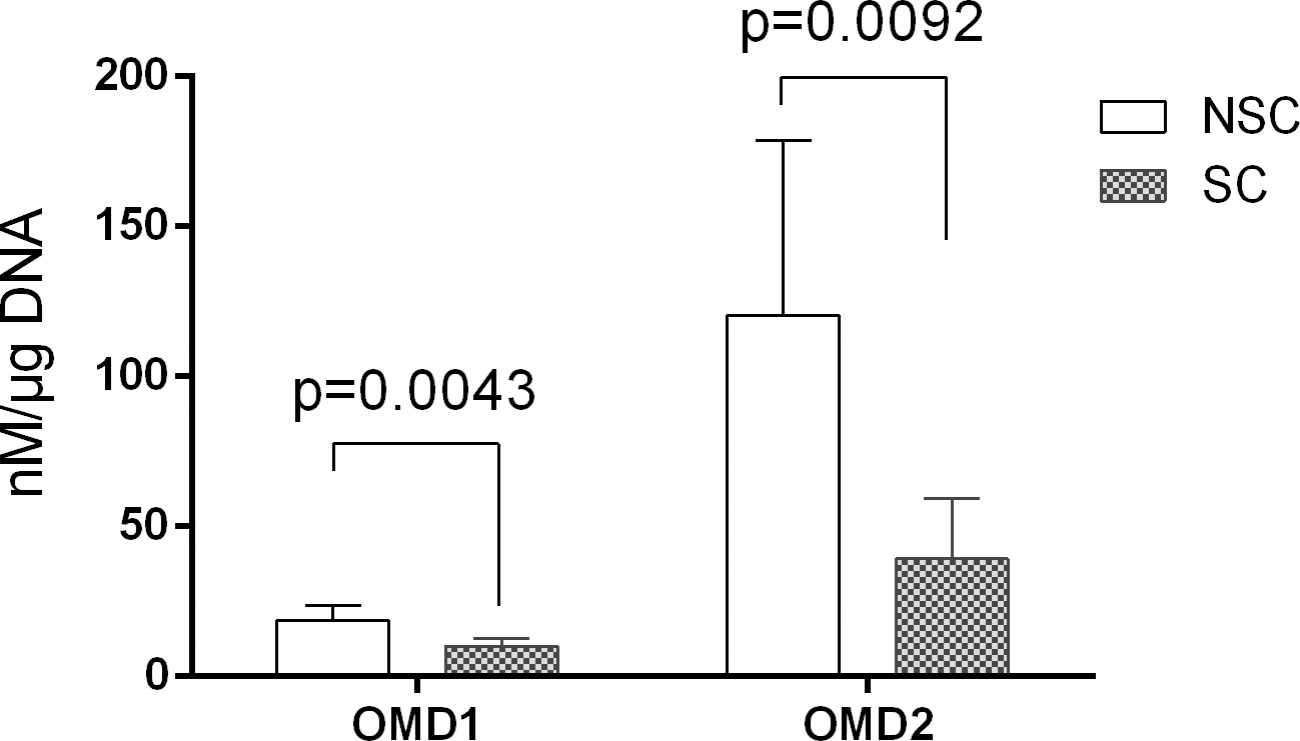
OMD1 and OMD2 levels found in osteoblasts cell culture supernatant. Data are means +/- SEM from 10 patients. Comparisons of mean values were performed by unpaired t-test.

To verify this hypothesis, we immunized Balb-C mice with a two peptides specific for OMD: ^148^LEHNNLEEFPFPLPK^162^ called OMD1 and ^261^LRMSHNKLQDIPYNI^276^ called OMD2. Using these antisera, competitive ELISAs were developed and were able to detect OMD in osteoblasts culture supernatant and human serum. These ELISAs confirming the decrease of OMD1 (0.54-fold, p = 0.0043) and OMD2 (0.33-fold, p = 0.0092) in SC compared to NSC secretome (Fig 3)

**Fig 4:**
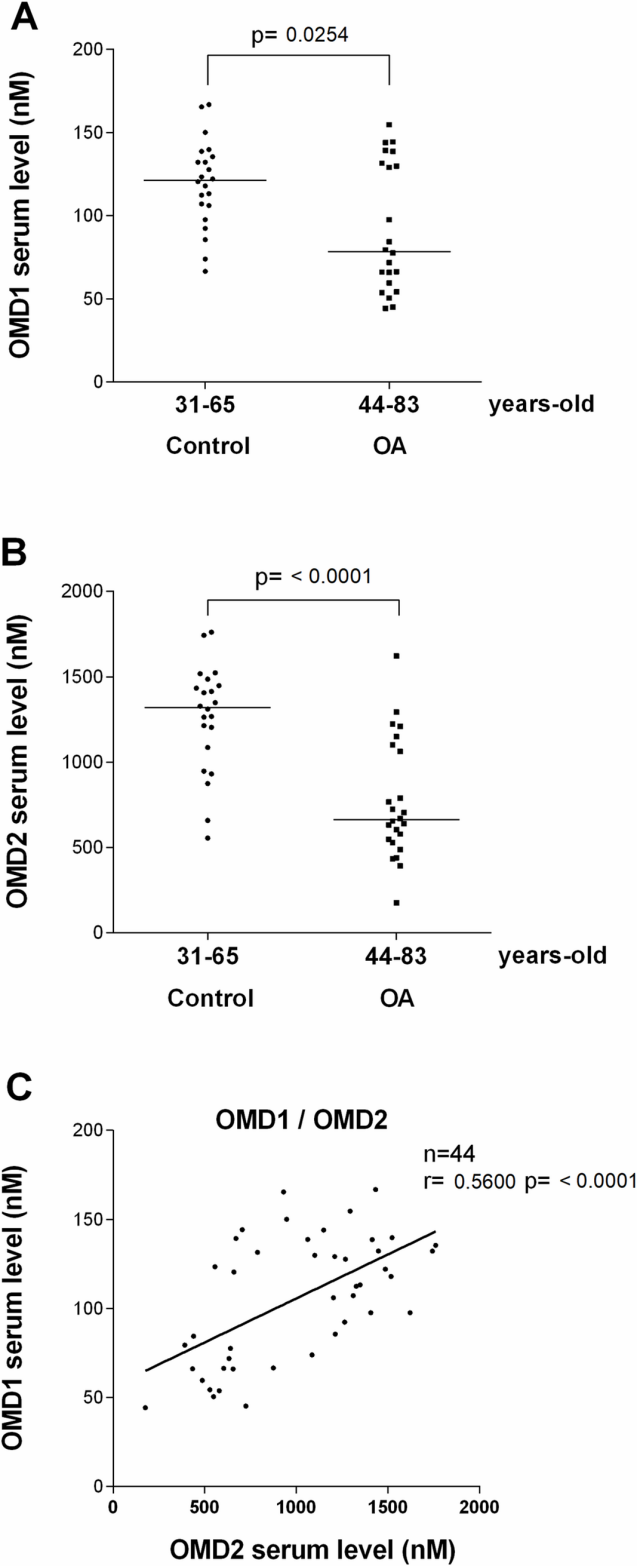
OMD1 and OMD2 levels found in healthy and OA human sera. (A) OMD1, (B) OMD2. Data are presented as scatter dot plots with median from 22 patients in each group. Comparisons of values were performed by Mann Whitney test. (C) Spearman correlation between OMD1 and OMD2 in serum, n = 44.

In human serum, we quantified OMD1 and OMD2 in 22 healthy persons aged from 31 to 65 year-old and 22 severe OA patients (before TKR) aged from 44 to 83 year-old. OMD1 and OMD2 were both significantly lower in OA compared to healthy patients (0.65-fold p = 0.0254 and 0.50-fold, p<0.0001, respectively, Fig 4). The OMD levels was not significantly different between men and women. The OMD1 level was significantly correlated with OMD2 level in healthy and OA sera (r = 0.56, p<0.0001, Fig 4C).

### Fibulin-3 fragment ELISA

**Fig 5:**
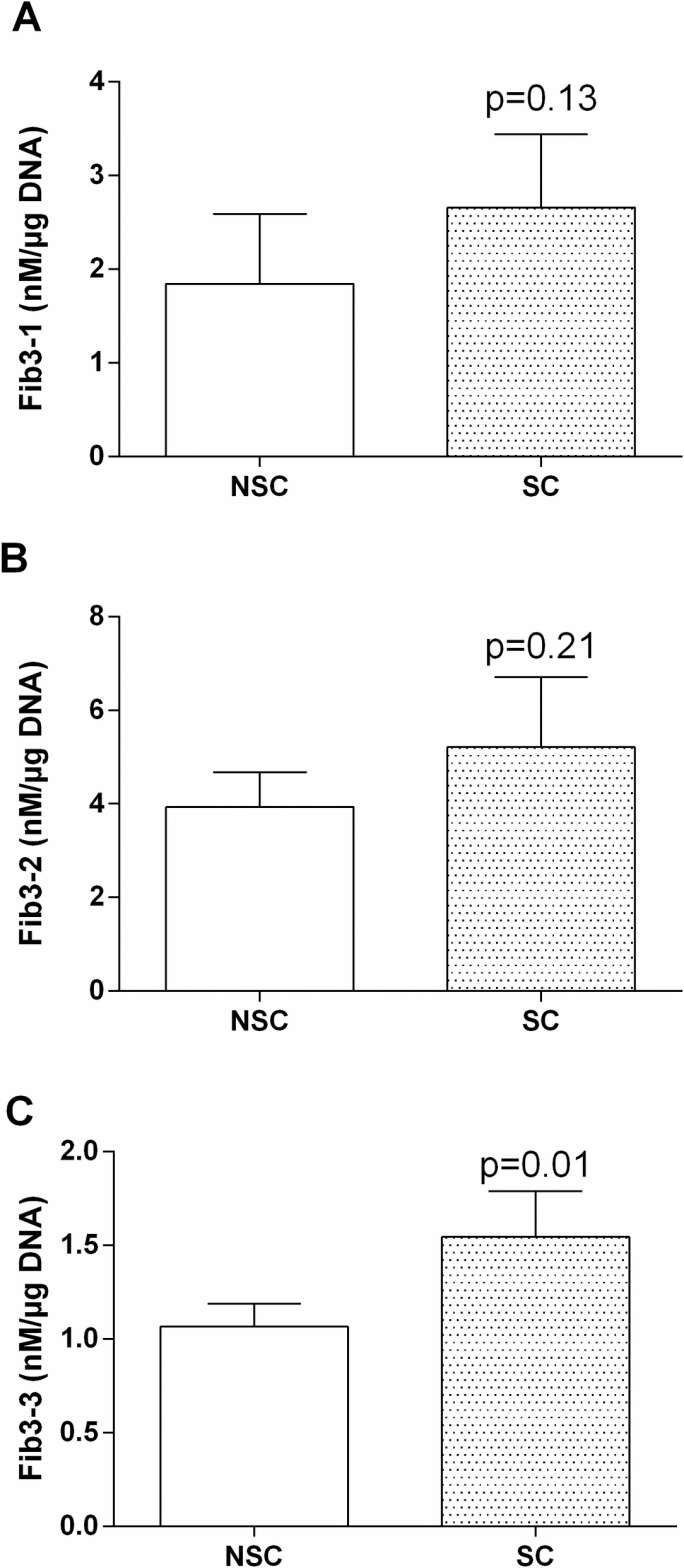
(A) Fib3-1, (B) Fib3-2 and (C) Fib3-3 levels found in osteoblasts cell culture supernatant. Data are means +/- SD from 4 patients for Fib3-1, 7 patients for Fib3-2 and 9 patients for Fib3-3. Comparisons of mean values were performed by paired t-test.

We have quantified three fibulin-3 fragments (Fib3-1, Fib3-2 and Fib3-3) in the supernatant of 10 independent osteoblasts cultures. Each culture was performed with cells coming from a single patient (Fig 5). The concentration of the fibulin-3 fragments was increased in SC osteoblasts supernatants compared to NSC, but the difference was only significant for Fib3-3 [Fib3-1 (1.44-fold p = 0.13, n = 4) Fib3-2 (1.32-fold p = 0.21, n = 7) Fib3-3 (1.45-fold p = 0.01, n = 9)].

## Discussion

So far, little information is available regarding the protein profiles disturbances in OA subchondral bone [15]. To extract and purify proteins from bone remains technically challenging [16]. Therefore, the in vitro study of osteoblasts secretome is an alternative, mainly if this study compares secretome of cells differently affected by the disease. For the first time, we have compared secretome of osteoblasts coming from non-sclerotic and sclerotic area of the same subchondral bone sample.

One hundred seventy five proteins, mainly extracellular matrix protein, were identified in NSC osteoblasts secretome. Among these extracellular matrix proteins some collagens, SPARC, lumican, fibronectin, decorin, biglycan, periostin, osteomodulin, vimentin were particularly abundant. Of course, the list of identified proteins is not exhaustive and some proteins are probably missing because minor proteins or small proteins are difficult to identify by mass spectrometry-based proteomic studies. The main explanations are the saturation of detectors with high abundance ions and the non-identification of small ionized peptides resulting of trypsin digestion.

Compared to NSC osteoblasts, 12 proteins were found in lower levels and 13 in higher levels in SC osteoblasts secretome (see Table 3). The majority of decreased proteins are involved in the mineralization process, like periostin [17], osteonectin [18], OMD, reticulocalbin and calumemin [19]. This finding could explain the lower mineralization of osteoid matrix in SC subchondral bone compared with NSC bone [3]. One well-documented explanation was the lower affinity of type I collagen for calcium [3], but our data also suggest that the decrease of some matrix proteins like periostin, osteonectin, OMD, calumenin and reticulocalbin could play a role.

The most down- regulated protein in SC osteoblasts was OMD. OMD, also called osteoadherin, is an extracellular matrix keratan sulfate proteoglycan member of the small leucine-rich repeat protein family. It was thought to be bone specific but recently OMD expression was found in articular chondrocytes and labrum-derived fibrochondrocytes [20]. Little is known about the biological activity of OMD. OMD is secreted into the matrix at the time of mineralization [21, 22]. The leucine-rich repeat motif of OMD is a dominant feature, comprising most of the core protein, while the acidic C terminus probably allows for binding to the hydroxyapatite of bone matrix [19]. In vitro overexpression OMD, treatment with recombinant OMD or RNA-mediated knockdown OMD in the mouse calvariae osteoblast cell line MC3T3E1 showed that the maturation state of osteoblasts and mineralization were increased by OMD [19]. It may be implicated in mineralization processes and could regulate osteoblast metabolism by binding alpha(V)beta(3)-integrin [21, 22]. Further, OMD regulates the extracellular matrix during bone formation, by controlling the diameter of type I collagen fibril [23]. Thus, a decrease of OMD secretion by SC osteoblasts could contribute to the abnormal mineralization of sclerotic bone in OA. [20]

Another key preliminary result of this study is the identification of different OMD forms in serum of OA patients. OMD has a molecular weight of 49.5 kDa but glycosylated forms of 60 to 85 kDa have been identified [24, 25]. In addition, human odontoblast may secrete a 110 kDa OMD form [22]. We have identified in subchondral bone osteoblasts supernatant a 70–75 kDa OMD form and in serum of OA patients a major 54 kDa form and some minor forms of 42, 35 and 30 kDa. These latter are probably fragments of the protein. Indeed, potential cleavage sites for MMP-2, -3 and -13 are present at residues 230 and 295 of the protein and the COOH terminal peptide could stay into the bone linked to hydroxyapatite [23]. One finding is that serum and osteoblasts secretome OMD have different molecular weight and were different from recombinant OMD. We explain this discrepancy by the level of OMD glycosylation in osteoblasts secretome and serum. Furthermore, the 54 kDa form is perhaps itself a fragment and not the full-length protein. Of course, these hypotheses must be verified. Interestingly, all these OMD forms are decreased in OA patients, suggesting that the decrease of OMD results of a decrease of the protein synthesis rather than an increase of OMD degradation.

The decrease of OMD in serum could reflect abnormal bone remodeling in OA subchondral bone suggesting that OMD could be a biomarker of subchondral bone metabolic changes in OA. Of course, this hypothesis has to be confirm in larger cohort including patients at different stages of the disease.

The second most enhanced protein in SC osteoblasts secretome compared to NSC osteoblasts secretome is Fibulin-3. Fibulin-3 is known to be involved in OA. This glycoprotein is present in the normal superficial layer of cartilage and decline with ageing [26]. The fragments, Fib3-1, Fib3-2 and Fib3-3 have been first identified in urine of OA patients [27] and found to be increased in OA serum [13]. Baseline fibulin-3 epitope concentrations are associated to the incidence of knee OA defined by the radiological and clinical ACR criteria among middle-aged overweight and obese women. In contrast, fibulin-3 epitopes are not associated with the knee joint space narrowing or the K&L score individually. This means that fibulin-3 epitopes predict more clinical than radiologic features in this population [13]. Fibulin-3 overexpression in a murine chondrocyte cell line ATDC5 induced changing in their cell morphology to spindle-shaped, increased their proliferative rate and suppressed their chondrogenic or hypertrophic differentiation, as assessed by aggrecan, type II and X collagen expression and alcian blue staining [28]. Recent experiments using siRNA or overexpressing fibulin-3 in human chondrocytes confirm that fibulin-3 has a role in maintaining the immature status of cells in the cartilage superficial layer [26]. Although fibulin-3 displays little interaction with elastin, fibrillin-1, and other elastic fiber or basement membrane components, mice deficient in fibulin-3 (fibulin-3 ^-/-^ mice) show defects in elastic fibers and microfibrils [29]. These fibulin-3 ^-/-^ mice also exhibit reduced fertility and lifespan accompanied with an early onset of ageing-associated phenotypes, including decreased body mass and bone density, generalized fat accumulation, and muscle and organ atrophy [29]. Few data are available on its precise role in bone. Further experiments are needed to precise the fibulin-3 role in bone metabolism, especially in OA subchondral bone sclerosis.

Among proteins displaying higher relative concentrations in SC samples, we found also CHI3L1 and CSF-1, two mediators controlling bone remodelling by increasing osteoclast differentiation [30, 31]. Silencing CHI3L1 with siRNA resulted in a significant decrease in bone resorption activity [30]. Several studies have reported increased levels of CHI3L1 protein and/or mRNA in patients with a wide spectrum of pathologies including rheumatoid arthritis, osteoarthritis (OA), giant cell arteritis and malignancies. Increased levels of CHI3L1 I was also reported in synovial fluid of knee OA patients compared to heatlhy subjects [32]. Moreover, it was observed that in patients with myeloma elevated serum concentrations of CHI3L1 aggravated bone destruction and were associated with an increase of bone resorption activity hastening the progression of bone disease [33]. CSF-1 is a cytokine that is required through all stages of osteoclast development. The osteoblasts are an important source of CSF-1 [31]. Mice lacking CSF-1 activity have an osteopetrotic phenotype due to a lack of osteoclasts [34]. Thus, the increase of CHI3L1 and CSF-1 could trigger the increase of bone remodelling observed in OA sclerotic subchondral bone.

Of course, our study suffer of some limitations: 1) the small number of subchondral bone samples which are probably not representative of all disease phenotype. This contributes to reduce the strength of our conclusion. 2) The use of subchondral bone samples collected at the late stage of disease before surgery for joint replacement. It would be interesting to investigate secretome of subchondral bone osteoblasts at different stage of the disease. 3) The culture model does not describe the ex-vivo behaviour of these cells, but rather the function of cells that grow out of the relevant tissues and then undergo extended culture before protein quantitation. It would be also interesting to investigate bone explant secretome to avoid interference with culture environment and cell differentiation occurring after cell outgrowth.

## Conclusions

We highlighted some proteins differentially secreted by the osteoblasts during subchondral sclerosis in OA. These changes could explain some features observed in OA subchondral bone, like increase of bone remodelling or decrease of bone matrix mineralization. Further, OMD and fibulin-3 could be biomarkers to follow metabolic changes in subchondral bone metabolism. Indeed, these proteins are strongly regulated in sclerotic bone and their concentrations are modified in serum of OA patients. Of course, this has to be confirm in animal models and in large cohort of OA patients but these first data are promising.
